# Supplementary figures and images for: ERK2 MAP kinase regulates SUFU binding by multisite phosphorylation of GLI1
Source: Life Sci Alliance. 2022 Jul 13;5(11):e202101353. doi: 10.26508/lsa.202101353 (PMC9279676; doi:10.26508/lsa.202101353)

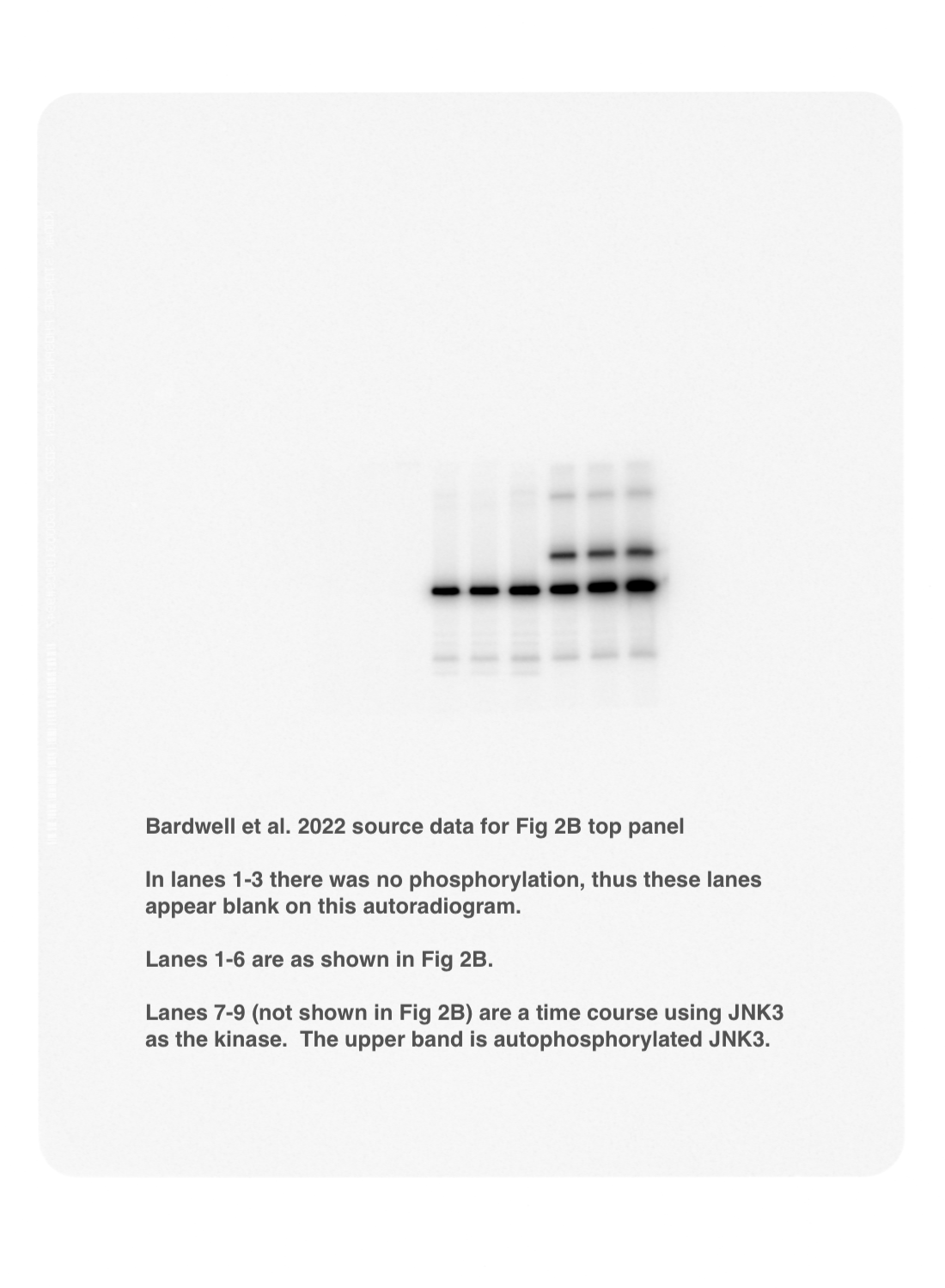

Supplement: Supplementary file 1 [file LSA-2021-01353_SdataF2.1.tif]

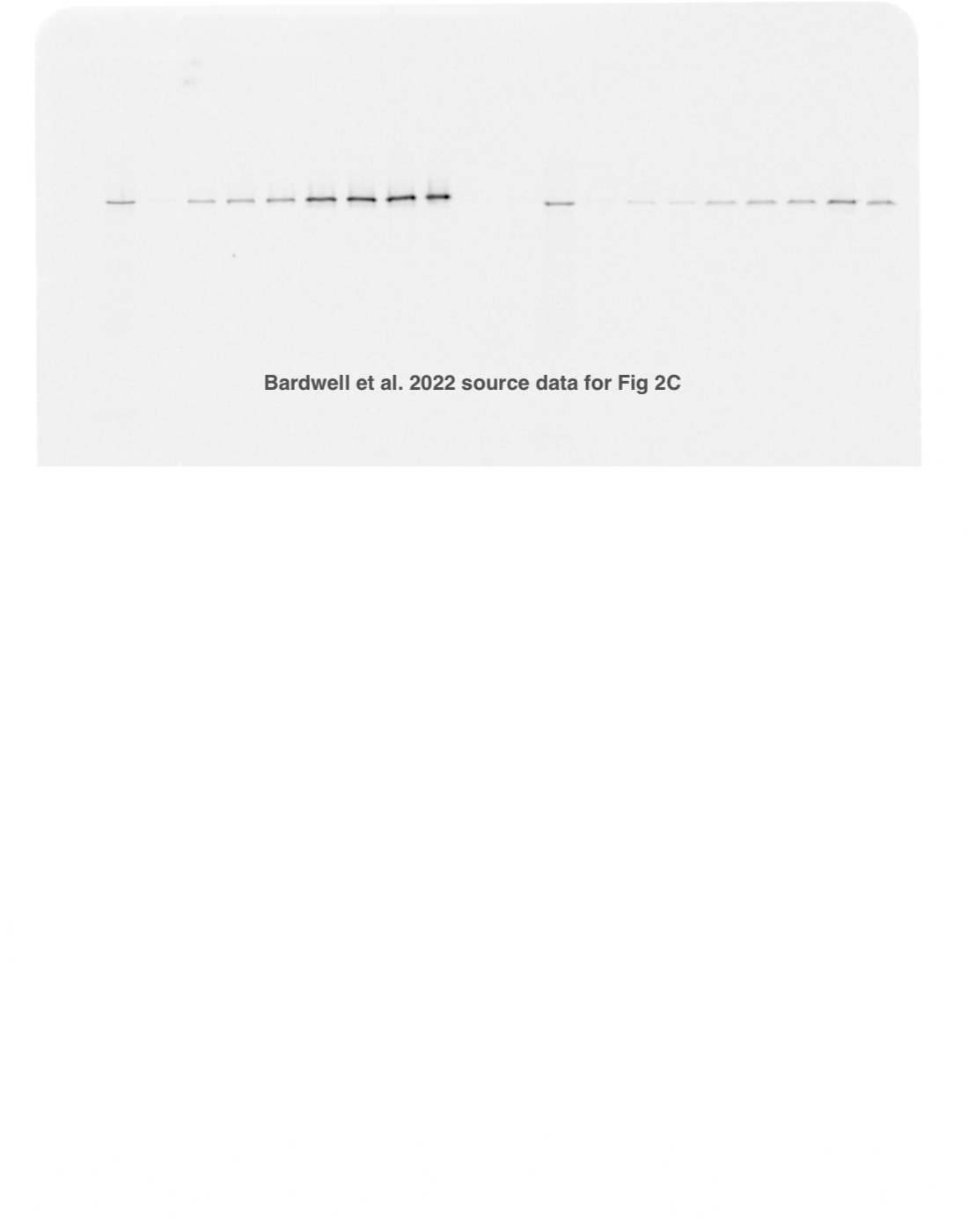

Supplement: Supplementary file 2 [file LSA-2021-01353_SdataF2.2.tif]

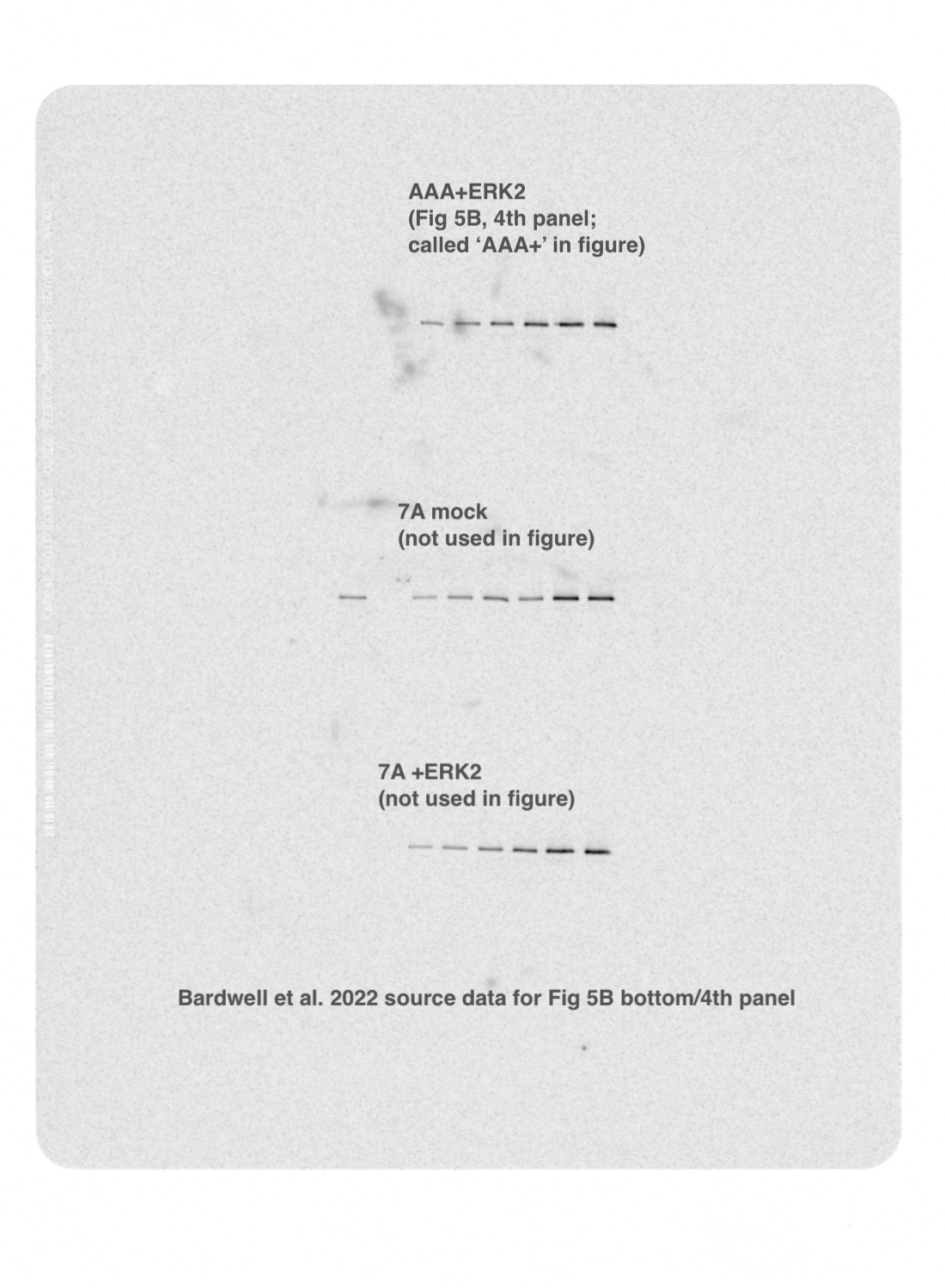

Supplement: Supplementary file 3 [file LSA-2021-01353_SdataF5.tif]

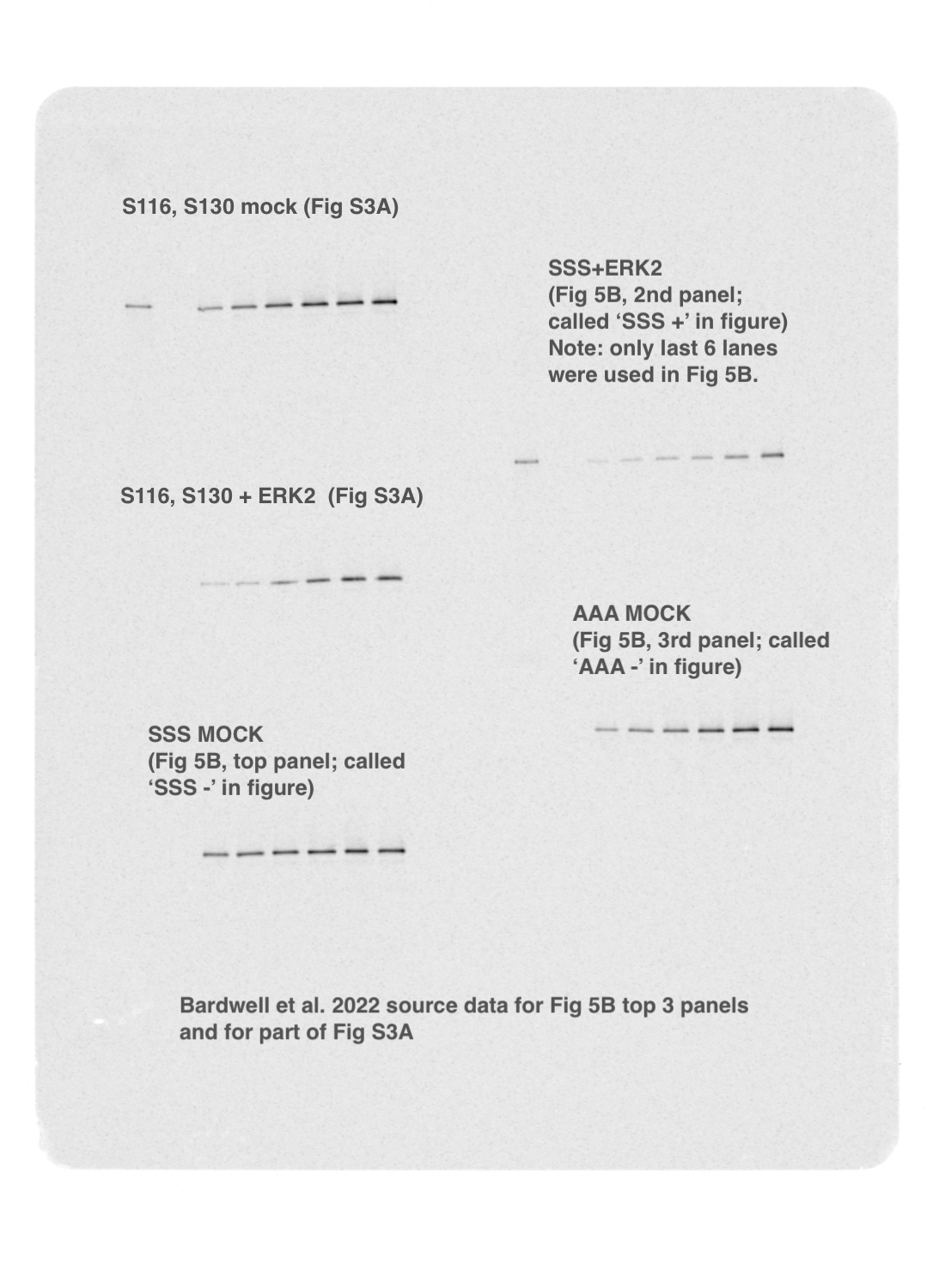

Supplement: Supplementary file 4 [file LSA-2021-01353_SdataF5_FS3.tif]

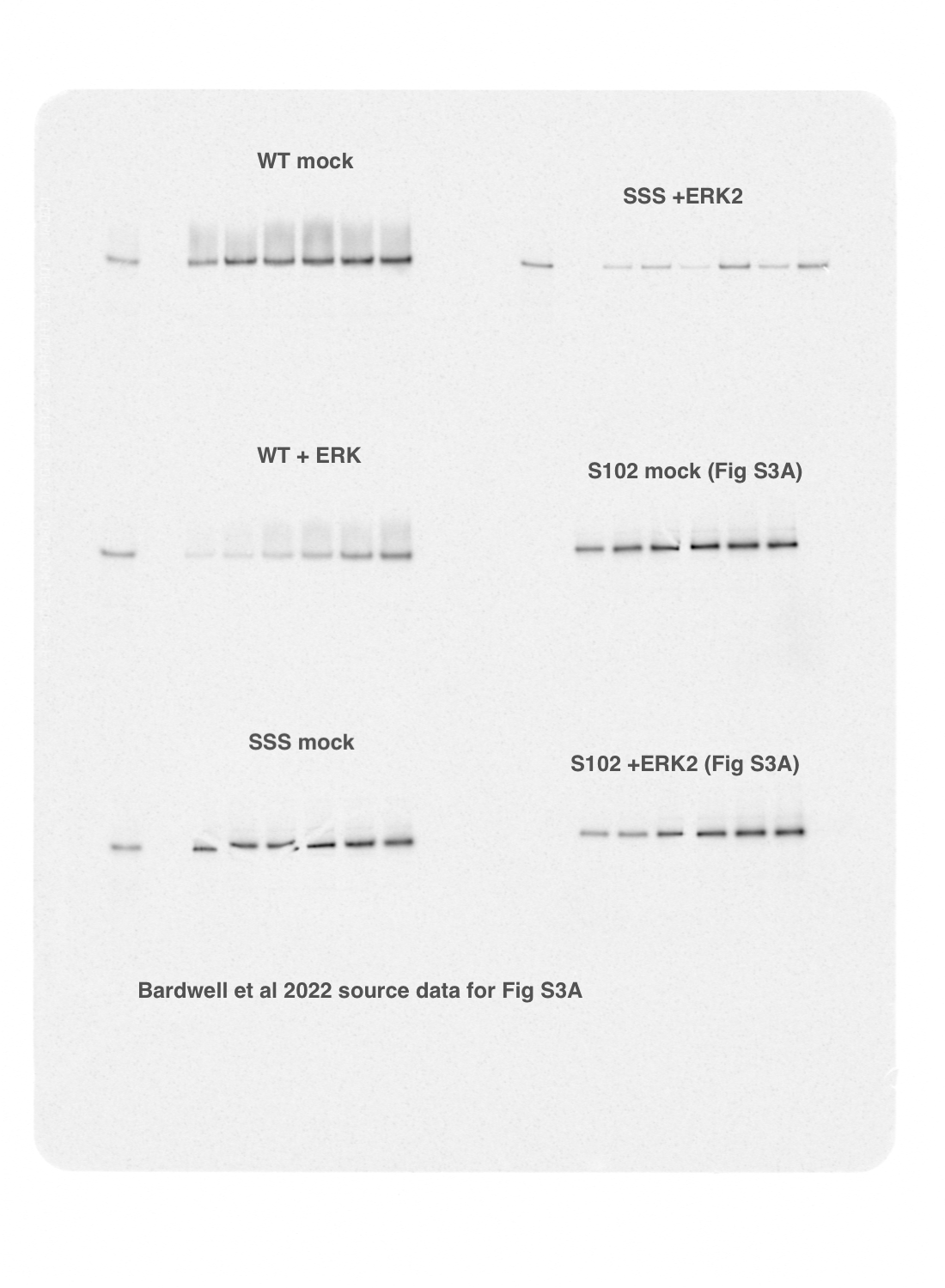

Supplement: Supplementary file 5 [file LSA-2021-01353_SdataFS3.1.tif]

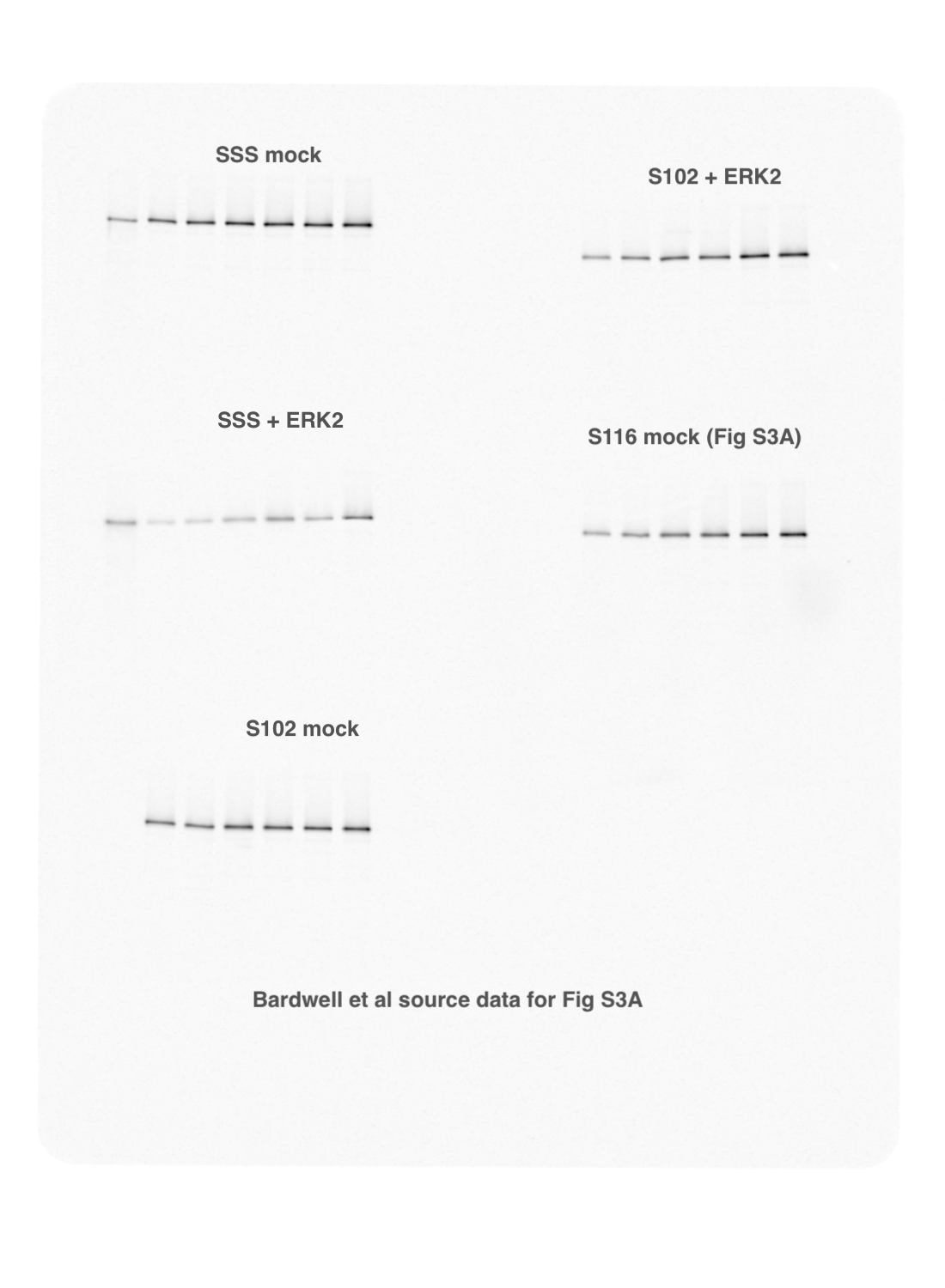

Supplement: Supplementary file 6 [file LSA-2021-01353_SdataFS3.2.tif]

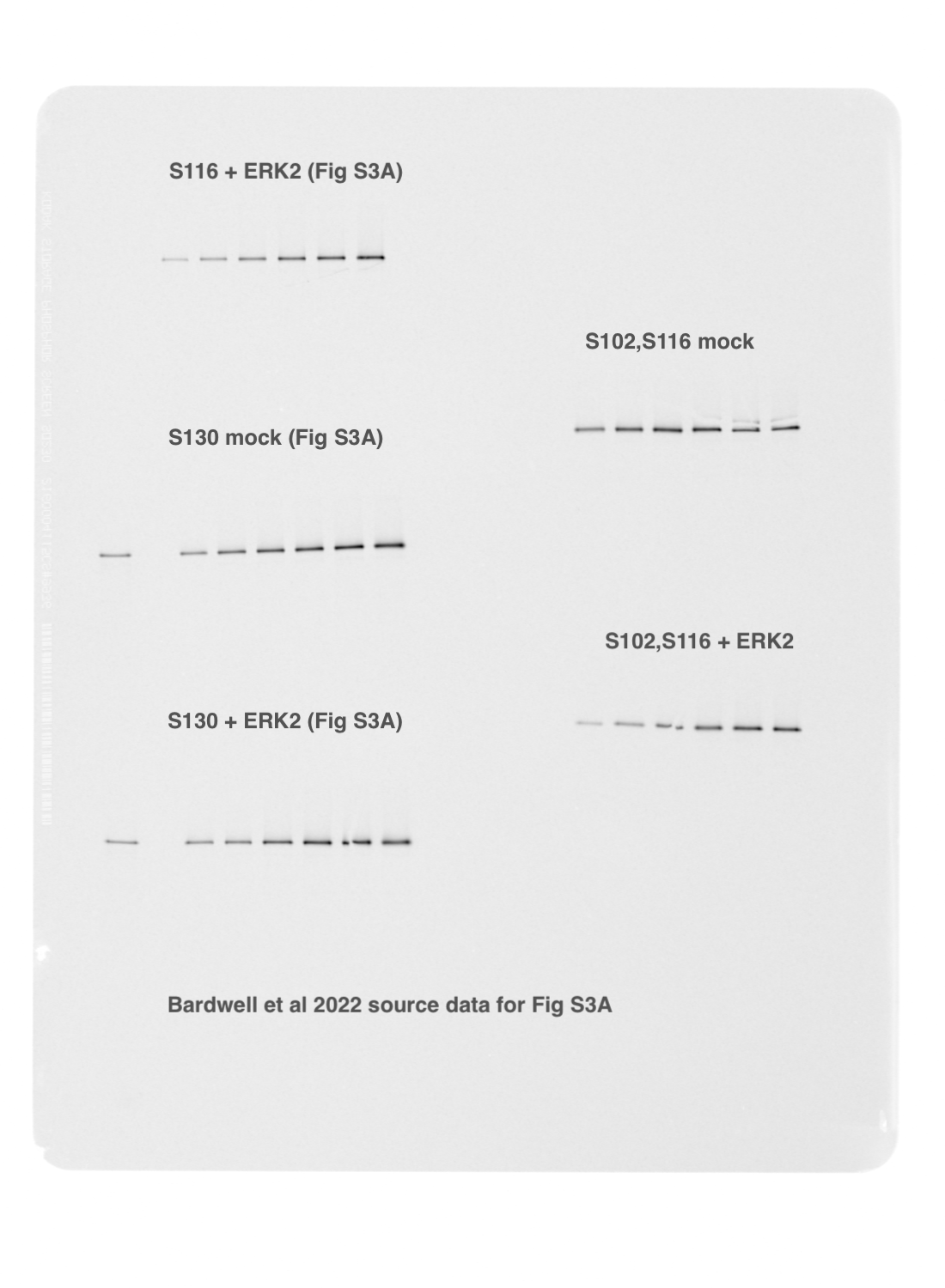

Supplement: Supplementary file 7 [file LSA-2021-01353_SdataFS3.3.tif]

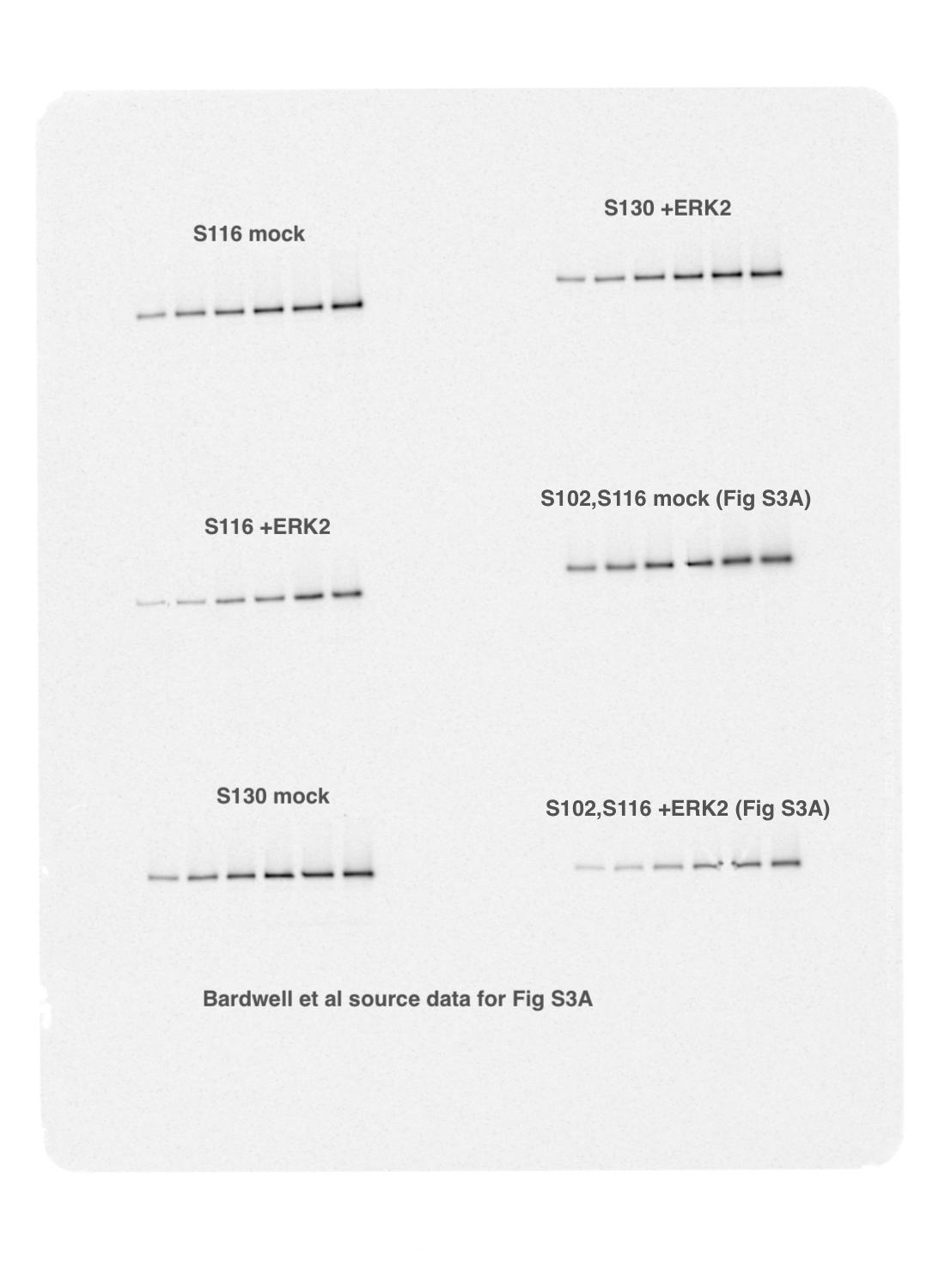

Supplement: Supplementary file 8 [file LSA-2021-01353_SdataFS3.4.tif]

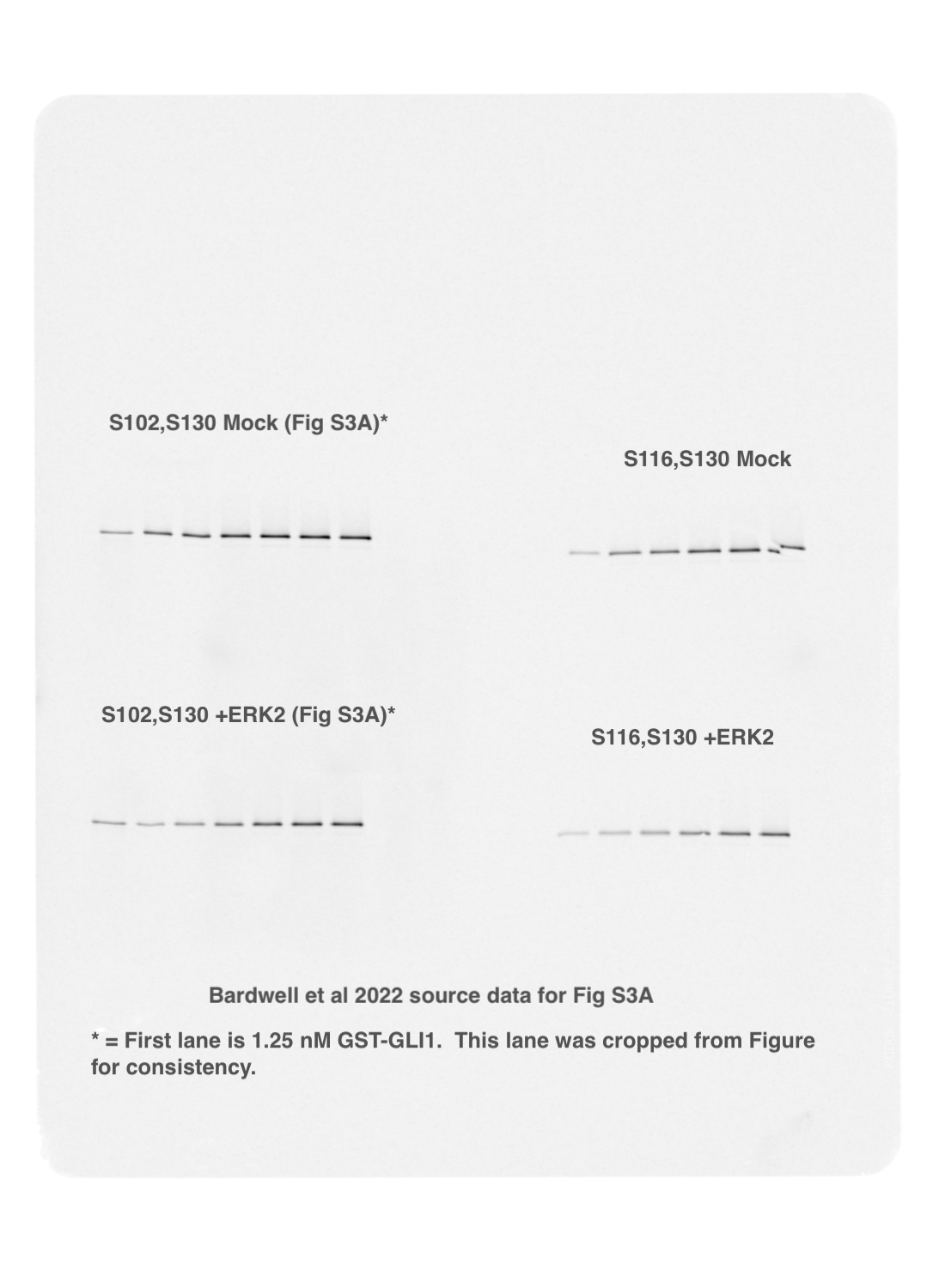

Supplement: Supplementary file 9 [file LSA-2021-01353_SdataFS3.5.tif]
